# *APOE* genotype modulates the impact of sleep duration on locus coeruleus functional connectivity in pre-clinical Alzheimer’s disease

**DOI:** 10.1093/braincomms/fcaf341

**Published:** 2025-09-09

**Authors:** Liang Gong, Haoyu Li, Maoxia Li, Yuan He, Duan Liu, Wen Zhou, Bei Zhang, Chunhua Xi

**Affiliations:** Department of Neurology, West China School of Medicine, Sichuan University, Sichuan University Affiliated Chengdu Second People’s Hospital, Chengdu, Sichuan 610017, China; Department of Neurology, Chengdu Medical College, Chengdu Second People’s Hospital, Chengdu, Sichuan 610017, China; Department of Neurology, Chengdu Medical College, Chengdu Second People’s Hospital, Chengdu, Sichuan 610017, China; Department of Neurology, Ya’an People’s Hospital, Chengdu Medical College, Ya’an, Sichuan 625000, China; Department of Neurology, North Sichuan Medical College, Chengdu Second People’s Hospital, Chengdu, Sichuan 610017, China; Department of Neurology, West China School of Medicine, Sichuan University, Sichuan University Affiliated Chengdu Second People’s Hospital, Chengdu, Sichuan 610017, China; Department of Neurology, West China School of Medicine, Sichuan University, Sichuan University Affiliated Chengdu Second People’s Hospital, Chengdu, Sichuan 610017, China; Department of Neurology, West China School of Medicine, Sichuan University, Sichuan University Affiliated Chengdu Second People’s Hospital, Chengdu, Sichuan 610017, China; Department of Neurology, The Third Affiliated Hospital of Anhui Medical University, Heifei, Anhui 230061, China

**Keywords:** locus coeruleus, functional connectivity, *APOE* genotype, sleep, Alzheimer’s disease

## Abstract

Sleep duration and Apolipoprotein E genotype are critical factors influencing Alzheimer’s disease progression. This study investigates the interaction between sleep duration and Apolipoprotein E genotype on the functional connectivity of the locus coeruleus in clinically unimpaired older adults with elevated amyloid beta, a population at risk for pre-clinical Alzheimer’s disease. The study included 692 clinically unimpaired older adults with elevated amyloid beta participants from the Anti-Amyloid Treatment in Asymptomatic Alzheimer’s Study (A4). Resting-state functional MRI data were analysed to construct locus coeruleus-based functional connectivity networks, and a 2 × 2 analysis of covariance was conducted to examine the main and interactive effects of sleep duration (normal versus short sleep) and Apolipoprotein E genotype (ɛ4− versus ɛ4+) on locus coeruleus-functional connectivity. Structural equation modelling was used to explore whether locus coeruleus-functional connectivity mediated the relationship between age and cognitive performance. Significant main effects of sleep duration and Apolipoprotein E genotype on locus coeruleus-functional connectivity were observed in the right temporal pole, middle cingulate cortex, and superior temporal gyrus. An interactive effect of sleep and Apolipoprotein E genotype was noted, influencing left locus coeruleus-functional connectivity in regions in the precentral gyrus, and right locus coeruleus-functional connectivity network in the middle temporal gyrus and lateral orbitofrontal cortex. Mediation analysis revealed that locus coeruleus-functional connectivity in the middle cingulate cortex and lateral orbitofrontal cortex partially mediated age associated cognitive decline. These findings suggest that locus coeruleus-functional connectivity networks, influenced by sleep duration and Apolipoprotein E genotype, play a crucial role in cognitive aging, particularly in memory function. Understanding these interactions may inform early intervention strategies to preserve cognitive health in older adults at risk for Alzheimer’s disease.

## Teaser

Gong *et al*. report that sleep duration and *APOE* genotype interact to modulate locus coeruleus function in older adults with elevated amyloid beta, affecting regions linked to memory and cognition. These findings highlight the role of sleep and genetic risk in early Alzheimer’s disease pathways, suggesting potential targets for intervention.

## Graphical Abstract

**Graphical Abstract:**
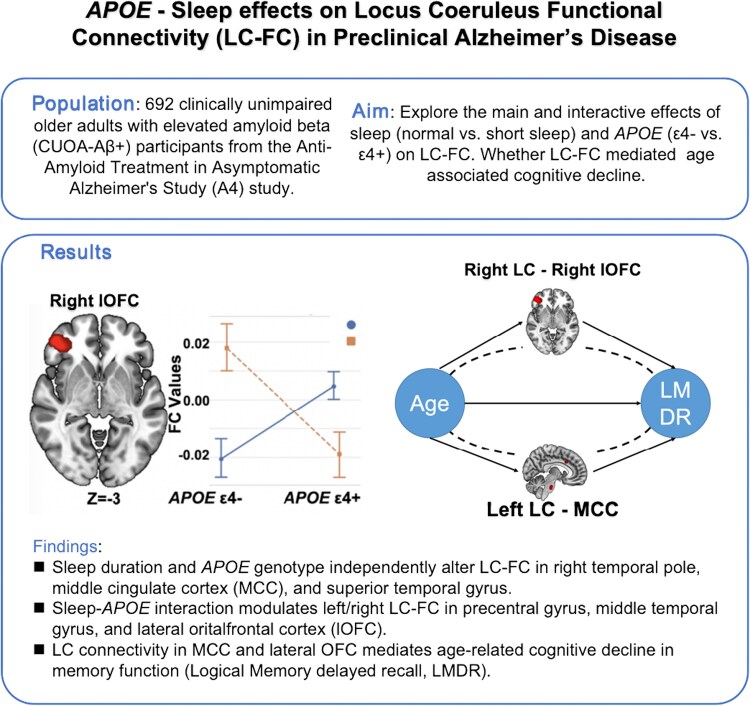

## Introduction

Alzheimer’s disease (AD), the leading contributor to cognitive impairment in older adults, manifests as a chronic neurodegenerative syndrome featuring insidious yet inexorable deterioration of higher-order mental faculties.^1,2^ Despite extensive research, the exact mechanisms driving AD, particularly in pre-clinical stages, remain unclear. During these early stages, individuals may appear cognitively unimpaired but exhibit neuropathological signs such as amyloid beta (Aβ) accumulation.^3,4^

The Apolipoprotein E (*APOE*) genotype, especially the *APOE* ɛ4 allele, is a significant genetic risk factor for sporadic AD.^5^ This allele is associated with increased amyloid deposition and an earlier onset of cognitive symptoms.^6^ Recent studies have further linked the *APOE* genotype to regional tau in the medial temporal lobe (including entorhinal and amygdala) and early neocortical regions, suggesting that amyloid may mediate the impact of the *APOE* genotype on tau in cognitively normal elders who are Aβ positive.^7,8^ In addition to genetic predisposition, lifestyle factors such as sleep have gained attention for their potential role in AD pathogenesis.^9,10^ Sleep disturbances are increasingly recognized as both a symptom and a risk factor for AD.^11^ Specifically, shortened sleep duration has been linked to greater amyloid deposition and accelerated cognitive decline in older adults.^9^ Furthermore, night time sleep duration has been associated with regional amyloid burden, especially in the anterior cingulate gyrus and medial orbitofrontal lobe, before significant Aβ deposition occurs.^12^ Previous research has demonstrated synergistic interactions between *APOE* genotype and sleep patterns in cognitive decline and AD pathogenesis.^13^ Experimental studies using AD transgenic mouse models have revealed that *APOE* ɛ4 genotype and sleep fragmentation cooperatively accelerate Aβ deposition while promoting both the initiation and progression of tau pathology.^14^ Furthermore, more consolidated sleep patterns appear to attenuate the association between *APOE* genotype and both AD incidence and tau neurofibrillary tangle deposition.^15^ However, the interaction between sleep duration and *APOE* genotype on brain function, particularly in clinically unimpaired older adults with elevated amyloid beta (CUOA-Aβ+), remains underexplored.

The locus coeruleus (LC), a brainstem nucleus, serves as the brain’s principal source of norepinephrine and plays a pivotal role in regulating arousal, attention and memory functions.^16,17^ It is also one of the first regions to exhibit tau pathology in the early stages of AD,^18^ suggesting its crucial involvement in the disease’s onset and progression. Our previous study has identified abnormal LC functional connectivity in the non-dementia stages of AD.^19^ The *APOE* ɛ4 allele may exacerbate tau neurotoxicity through multiple mechanisms, including the inhibition of noradrenaline transport in the LC in AD mouse models,^20,21^ as well as altered functional connectivity between the LC and key cortical regions, such as the occipital cortex and precuneus, during the pre-clinical stages of AD.^22^ Additionally, abnormalities in the LC-norepinephrine system have also been observed in patients with chronic insomnia disorder.^23,24^ These findings underscore the necessity of exploring the combined impact of genetic and lifestyle factors on the LC network, which may significantly influence cognitive outcomes in aging.

The present investigation seeks to elucidate the interactive mechanisms by which sleep duration and *APOE* genotype jointly modulate early stage cognitive decline and AD pathogenesis. Our objectives include: (i) Characterizing the epistatic relationship between diurnal sleep patterns and *APOE* ɛ4 carrier status on LC functional connectivity in CUOA-Aβ+ individuals and (ii) Exploring the possible mediating role of the LC network in age-related cognition decline. We hypothesize that shorter sleep duration and the *APOE* ɛ4 allele interacts to disrupt LC functional connectivity, and that this disruption may mediate age-related cognitive decline in CUOA-Aβ+ individuals.

## Materials and methods

### Participants

The current investigation sourced participants from the A4 study cohort, a landmark secondary prevention trial targeting pre-clinical AD (ClinicalTrials.gov: NCT02008357). Building upon established inclusion paradigms,^25^ subjects met stringent selection criteria: (i) clinical unimpaired status (CDR = 0) with preserved activities of daily living; (ii) biomarker-confirmed amyloidosis ^[18F]^florbetapir; (iii) neuropsychometric profile within normal ranges (Mini-Mental State Examination (MMSE) 25–30, Logical Memory delayed recall (LMDR) 6–18); (iv) multimodal neuroimaging data (3D sMRI and rs-fMRI). Sleep phenotyping incorporated based on self-reported data, classifying sleep duration as normative (7–8 h) or restricted (≤6 h).^9^  *APOE* genotyping was performed, with ɛ4 carrier status defined as the presence of ≥1 copy of the risk allele. Accordingly, the *APOE* ɛ4 + group comprised ɛ4/ɛ4 and ɛ3/ɛ4 genotypes, while the *APOE* ɛ4− group included ɛ3/ɛ3 and ɛ2/ɛ3 genotypes. The study was conducted in accordance with the Declaration of Helsinki. Participants provided written informed consent. All research activities were approved by Institutional Review Boards at the participating study sites. The complete A4 study site list is available at https://atri.usc.edu/study/a4-study/#lists.

### Neuropsychological test

Cognitive performance was assessed using the MMSE and Pre-clinical Alzheimer’s Cognitive Composite (PACC) score^26,27^ to assess general cognition, digit symbol substitution test (DSST) to measure executive function, LMDR test and free and cued selective reminding test (FCSRT)^28^ to assess memory performance. All cognitive performance measures were adjusted for age and education in the statistical analyses.

### Neuroimaging methods

Amyloid status ([18F]florbetapir) was assessed using a hybrid quantitative/qualitative method from the A4 Study.^25^ Aβ+ was defined as a mean cortical standardized uptake value ratio ≥1.10.

The detailed MRI parameters were shown in the A4 study website (https://www.a4studydata.org/). The rs-fMRI images were pre-processed using SPM12 (http://www.fil.ion.ucl.ac.uk/spm) and DPABI 7.0 (http://rfmri.org/dpabi), implemented in MATLAB version 9.0 (The MathWorks, Inc., Natick, MA, USA).^29^ Pre-processing involved: discarding initial five volumes; slice timing correction; realignment; spatial normalization to 3 × 3 × 3 mm voxels; 0.01–0.1 Hz low-pass filtering; detrending; covariate regression (24 motion parameters, WM, CSF, and global signals); and 6-mm Gaussian smoothing. Total intracranial volume (ICV) was included as a covariate to control regional volumetric effects.^30^ Due to the small size of the LC nucleus, we adopted a 3-mm Gaussian smoothing method for sensitivity analysis.

### LC functional network construction

LC-functional connectivity (FC) analysis was conducted in DPABI using left and right LC seeds from the AAL3 atlas.^31^ The LC mask from the AAL3 atlas, originally defined in a high-resolution (0.5 mm³) MNI152 template, was non-linearly transformed into the colin27 single-subject T1 template using white matter segments as reference to ensure accurate registration across different MNI spaces. The LC has been used as a seed region in multiple prior studies due to its well-defined anatomical location and functional significance.^19,23,32^ As previous studies found different FC patterns between left and right LC,^19,23,32,33^ we used the left LC and right LC separately as seeds in the present study. Whole-brain voxel time courses were correlated with LC signals via Pearson’s correlation and Fisher’s Z-transformed.^34,35^ Framewise displacement (FD) was covaried, and subjects exceeding 3 mm translation or 3° rotation were excluded. Moreover, we used the scrubbing method to censor the bad images with FD >0.5, and one image before and two images after the bad image were deleted.

### Statistical analyses

Statistical analyses used SPSS 24.0 and MATLAB 8.0. Demographic/cognitive comparisons employed 2 × 2 ANCOVA (Sleep × *APOE* genotype) covarying for sex, age, and education years. Two sample *t*-test was used to explore the potential difference between *APOE* ɛ4/ɛ4 and ɛ3/ɛ4 groups. Age-cognition relationships were assessed via partial correlations (controlling sex/education), with *P* < 0.05 significance.

One sample *t*-tests were used to present the left and right LC-FC network patterns for each group (Supplementary Fig. 1, *P* < 0.05, uncorrected). Voxel-wise LC-FC maps underwent 2 × 2 ANCOVA (Sleep × *APOE*) with sex/age/education/ICV/FD as covariates. Cluster significance required voxel *P* < 0.005, Gaussian random field (GRF)-corrected cluster *α* < 0.01 (minimum 50 voxels). FC values from significant clusters were extracted for mediation/correlation analyses. Partial correlations assessed cognitive-LC-FC associations in cognitively normal Aβ+ older adults (covariates: sex/education). The *post-hoc* analyses of ANCOVA results for neuroimaging data between *APOE* ɛ4/ɛ4 and ɛ3/ɛ4 groups were conducted by two sample *t*-test.

To explore the complex inter-relationships among age, the LC-FC network, and cognitive performance, Structural Equation Modelling (SEM) was employed to investigate the mediation effects of brain regions (including the main and interaction effects of sleep and *APOE* genotype) on the relationship between age and cognitive performance (measured by PACC, MMSE, DSST, FCSRT, and LMDR scores). The mediation analysis involved calculating both the direct effects (*c* path) of age on cognitive outcomes and the indirect effects (*a* * *b* paths) via brain regions. The proportion of the total effect mediated by each brain region was calculated using the following formula:


$$\mathrm{Proportion}\;\mathrm{Mediated}=\mathrm{Indirect}\;\mathrm{Effect}(a^{*}b)/\mathrm{Total}\;\mathrm{Effect}(c)\times 100$$

^36^

The model was controlled for education and sex. Data were analysed using the ordinary least squares regression approach within the SEM framework. The 95% confidence intervals were determined using bias-corrected non-parametric bootstrapping with 1000 resamples. SEM analysis was conducted in Python using the ‘statsmodels’ library, focusing on assessing both the significance of individual paths and the overall fit of the model. This study adopted an uncorrected analytical approach to maximize sensitivity in detecting potential neural pathways.

## Results

### Sample characteristics

Table 1 summarizes demographic/cognitive data for 692 CUOA-Aβ+ participants (including 501 normal sleep, 402 *APOE* ɛ4 + carriers, and 487 females). No group differences in sex, age, or education between sleep duration subgroups. *APOE* ɛ4 + carriers showed younger mean age versus ɛ4− carriers (*P* < 0.05). *APOE* ɛ4 + had lower FCSRT scores than ɛ4− (*F* = 4.48, *P* = 0.035, age/education-adjusted). No significant effects of *APOE* genotype on other cognitive measures. No sleep × *APOE* interactions in demographic/cognitive comparisons.

**Table 1:** Demographic and neuropsychological data for all groups

	Normal sleep duration (*n* = 501)			Short sleep duration (*n* = 191)			*APOE* ɛ4−(*n* = 290)	*APOE* ɛ4 + (*n* = 402)	Sleep effect *F(p)*	*APOE* effect *F(p)*^a^	Sleep^a^*APOE* effect *F(p)*^a^
	*APOE* ɛ4− (*n* = 210)	*APOE* ɛ4+ (*n* = 291)	ALL	*APOE* ɛ4− (*n* = 80)	*APOE* ɛ4+ (*n* = 111)	ALL					
Sex (F/M)	132/78	180/111	312/189	43/37	61/50	104/87	175/115	241/161	0.32 (0.06)^a^	0.02 (0.91)^a^	0.88 (0.15)^a^
Age (yrs)	72.54 ± 4.96	71.32 ± 4.54	71.83 ± 4.76	73.70 ± 4.95	71.14 ± 4.06	72.27 ± 4.41	72.89 ± 5.08	71.28 ± 4.49	1.48 (0.22)	22.22 (<0.001)	2.74 (0.98)
Education (yrs)	16.62 ± 2.99	17.17 ± 2.64	16.50 ± 2.76	16.12 ± 2.81	16.25 ± 2.64	16.43 ± 3.06	16.56 ± 3.07	16.43 ± 2.72	0.14 (0.71)	0.07 (0.78)	0.31 (0.57)
MMSE	28.77 ± 1.29	28.65 ± 1.25	28.70 ± 1.32	28.75 ± 1.29	28.51 ± 1.33	28.61 ± 1.31	28.76 ± 1.27	28.71 ± 1.27	0.46 (0.50)	2.45 (0.12)	0.26 (0.61)
PACC	−0.23 ± 2.80	−0.61 ± 2.85	−0.45 ± 2.84	−0.17 ± 2.39	−0.64 ± 2.48	−0.45 ± 2.45	−0.36 ± 2.72	−0.44 ± 2.67	0.003 (0.95)	3.21 (0.74)	0.05 (0.83)
DSST	42.01 ± 8.59	42.40 ± 9.21	42.24 ± 8.95	41.91 ± 8.44	42.36 ± 9.17	42.17 ± 8.85	42.04 ± 8.76	43.23 ± 9.02	0.008 (0.93)	0.29 (0.58)	0.001 (0.97)
FCSRT	76.14 ± 5.94	75.16 ± 6.42	75.57 ± 6.32	76.56 ± 5.69	75.31 ± 5.91	75.83 ± 5.84	76.26 ± 5.87	75.20 ± 6.28	0.29 (0.59)	4.48 (0.035)	0.07 (0.79)
LMDR	11.79 ± 3.52	11.30 ± 3.36	11.50 ± 3.43	11.85 ± 3.48	11.48 ± 3.25	11.63 ± 3.35	11.56 ± 3.46	11.29 ± 3.21	0.17 (0.68)	2.15 (0.14)	0.40 (0.84)
ICV (ml)	1514.45 ± 145.10	1535.15 ± 1150.09	1526.52 ± 148.24	11542.63 ± 177.89	1527.17 ± 151.61	1533.65 ± 162.85	1522.28 ± 155.10	1532.95 ± 1150.36	0.59 (0.44)	0.04 (0.84)	1.89 (0.17)

^a^Logistic regression analysis was used.

*APOE*, Apolipoprotein E gene; PACC, pre-clinical Alzheimer’s cognitive composite; DSST, digit symbol substitution test; FCSRT, free and cued selective reminding test; LMDR, logical memory delayed recall; MMSE, mini-mental state examination; ICV, intracranial volume.

The *APOE* ɛ3/ɛ4 group was significantly older than the *APOE* ɛ4/ɛ4 group (*P* = 0.002). *Post-hoc* analysis of demographic and cognitive measures revealed no other significant differences between the two groups (Supplementary Table 1, all *P* > 0.05).

Age was negatively associated with cognitive performance, including general cognition (MMSE, *r* = −0.28, *P* = 6.31*10^−6^; PACC, *r* = −0.44, *P* = 1.77*10^−20^), memory performance (LMDR, *r* = −0.17, *P* = 3.06*10^−3^; FCSRT, *r* = −0.35, *P* = 1.42*10^−11^), and executive function performance (DSST, *r* = −0.44, *P* = 2.07*10^−22^).

### The main effect of sleep and *APOE* genotype on the LC-FC network

**Figure 1:**
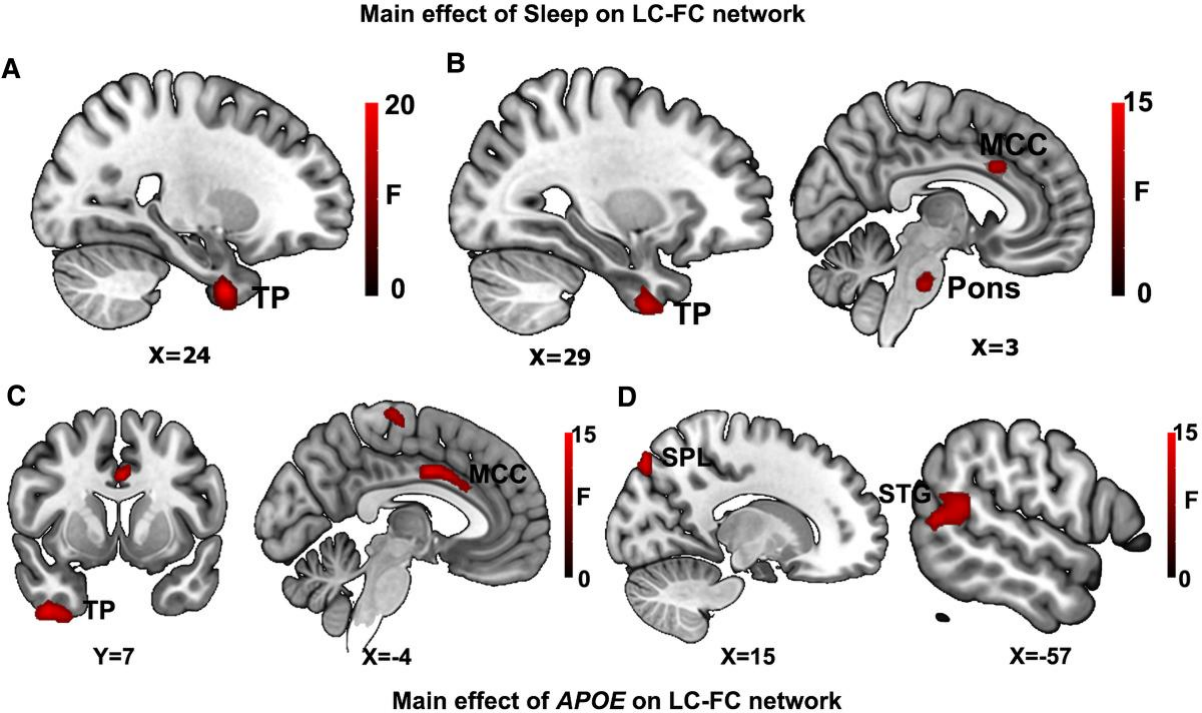
**The main effects of sleep duration and *APOE* genotype on the left and right LC-FC network (*P* < 0.005, *α* < 0.01, GRF correction, *n* = 692).** Voxel-wise ANCOVA results revealed significant main effects of (1) sleep duration on left LC-FC (**A**) and right networks (**B**) and (2) *APOE* genotype on left LC-FC (**C**) and right networks (**D**). The colour bar means the *F*-value. LC-FC, locus coeruleus functional connectivity; TP, temporal pole; MCC, middle cingulate cortex; STG, superior temporal gyrus; SPL, superior parietal lobule.

As illustrated in Table 2 and Fig. 1, in the left LC-FC network, sleep duration significantly modulated the right temporal pole (TP), *APOE* genotype differentially influenced left middle cingulate cortex (MCC) and the right TP. In the right LC-FC network, sleep duration modulated the right TP, the pons, and the bilateral MCC, *APOE* genotype influenced the left superior temporal gyrus (STG) and the right superior parietal lobule (SPL).

**Table 2:** Brain areas with significant effects of sleep duration, APOE and sleep × APOE on the LC-FC network

	Brain regions	Brodmann area	Cluster size (voxels)	MNI coordinates (*x*, *y*, *z*)	Peak *F*-score
Left LC-FC network					
Main effect of sleep	Right TP	36	129	30, 3, −42	23.68
Main effect of *APOE*	Left MCC	24	91	−3, −3, 36	16.10
	Right TP	20	109	42, 6, −48	14.22
Interactive effect of Sleep × *APOE*	Right PreC	4	63	18, −30, 57	13.06
Right LC-FC network					
Main effect of sleep	Right TP	21	68	30, 3, −48	13.02
	Right Pons	-	58	12, −21, −33	18.31
	Bilateral MCC	24	118	−3, 12, 33	14.85
Main effect of *APOE*	Left STG	22	82	−60, −51, 15	12.69
	Right SPL	7	53	15, −81, 51	13.82
Interactive effect of sleep × *APOE*	Left MTG	2	117	−48, −9, −27	15.42
	Right lOFC	47	109	45, 45, −3	20.00

LC-FC, locus coeruleus functional connectivity; TP, temporal pole; MCC, middle cingulate cortex; PreC, precentral gyrus; STG, superior temporal gyrus; SPL, superior parietal lobule; MTG, middle temporal gyrus; lOFC, lateral orbitofrontal cortex.

### The interactive effect of sleep and *APOE* genotype on the LC-FC network

**Figure 2:**
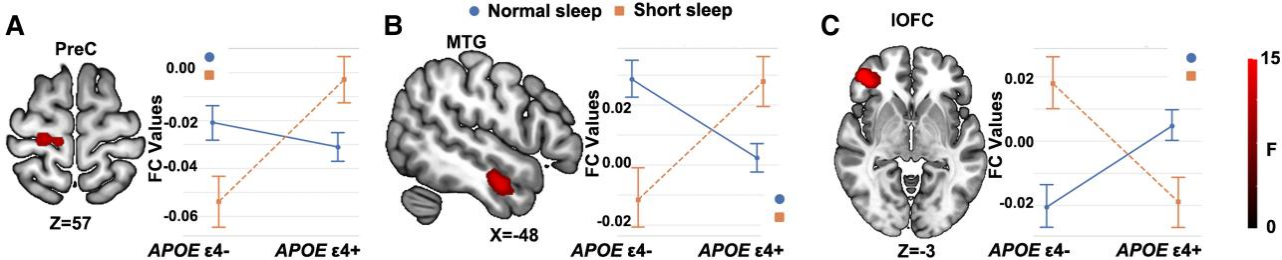
**Voxel-wise ANCOVA results revealed significant interactive effects of sleep duration and *APOE* genotype on the left LC-FC (A) and right LC-FC (B and C) networks (*P* < 0.005, *α* < 0.01, GRF correction, *n* = 692).** The colour bar means the *F*-value. LC-FC, locus coeruleus functional connectivity; PreC, precentral gyrus; MTG, middle temporal gyrus; lOFC, lateral orbitofrontal cortex.

As shown in Table 2 and Fig. 2, the interactive effect of sleep and *APOE* genotype on the left LC-FC network was observed in the right precentral gyrus (PreC). Compared to *APOE* ɛ4− individuals, those with the *APOE* ɛ4 + genotype showed lower LC-FC in the normal sleep group and higher LC-FC in the short sleep group. The interactive effect of sleep and *APOE* genotype on the right LC-FC network was found in the left middle temporal gyrus (MTG) and right lateral orbitofrontal cortex (lOFC). In the MTG of the right LC-FC network, individuals with *APOE* ɛ4 + showed lower LC-FC in the normal sleep group and higher LC-FC in the short sleep group, compared to those with *APOE* ɛ4−. In the lOFC of the right LC-FC network, *APOE* ɛ4 + individuals exhibited higher LC-FC in those with normal sleep and lower LC-FC in those with short sleep, relative to *APOE* ɛ4− individuals.

*Post-hoc* analysis of the neuroimaging ANCOVA results revealed no significant differences between the *APOE* ɛ4/ɛ4 and ɛ3/ɛ4 groups (Supplementary Table 2, all *P* > 0.05).

### The relationship between the LC-FC network and cognitive performance in CUOA-aβ+

Partial correlations revealed sleep-modulated LC functional connectivity associations with memory/executive functions in CUOA-Aβ+ individuals, exclusively observed in the short sleep subgroup. Notably, left LC-right TP connectivity positively correlated with LMDR scores (*r* = 0.16, *P* = 0.023) within this cohort. No additional associations between LC-FC networks and cognitive domains reached statistical significance. However, none of the correlations remained significant after correction for multiple comparisons.

### The mediation analysis results

**Figure 3:**
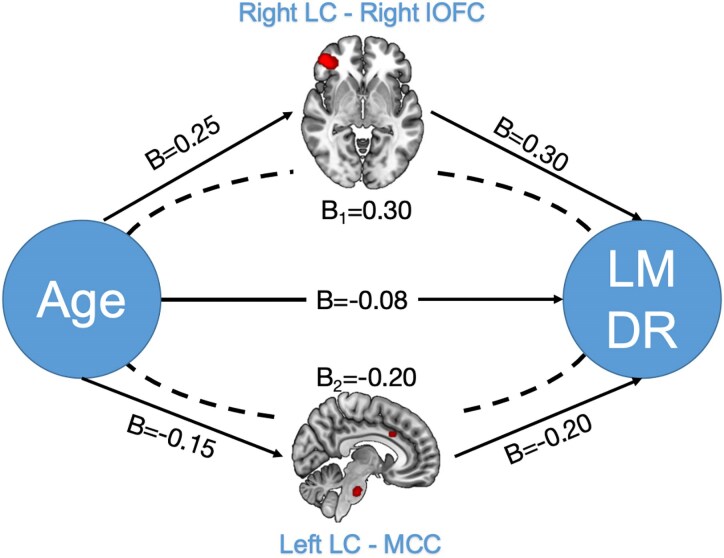
**Mediation analysis demonstrated that the LC-FC network mediates the relationship between age and memory performance in CUOA-Aβ+ individuals (*P* < 0.05, uncorrected, *n* = 692).** LC-FC, locus coeruleus functional connectivity; CUOA-Aβ+, clinically unimpaired older adults with elevated amyloid beta; LMDR, logical memory delay recall performance; lOFC, lateral orbitofrontal cortex; MCC, middle cingulate cortex.

The SEM model showed that two brain regions in the LC-FC network mediated the relationship between age and memory performance in CUOA-Aβ+ individuals. As shown in Fig. 3, the FC between the right LC and right lOFC partially mediated the relationship between age and LMDR scores. The indirect effect of age on LMDR via lOFC was significant (*B* = 0.30, *P* = 0.037), accounting for ∼12.8% of the total effect. The FC between the left LC and bilateral MCC also acted as a significant mediator in the relationship between age and LMDR scores. The indirect effect via MCC was significant (*B* = −0.20, *P* = 0.021), accounting for 14.3% of the total effect.

### Sensitivity analysis results

Due to the small size of the LC nucleus, we adopted a 3 mm Gaussian Kernel smoothing method for sensitivity analysis. The results were presented in Supplementary Fig. 2. The main effect of *APOE* genotype, sleep and their interactive effect on the LC-FC Network were similar with 6-mm Gaussian Kernel smoothing method.

## Discussion

The current study sought to investigate the interaction between sleep duration and *APOE* genotype on the left and right LC-FC networks in CUOA-Aβ+ individuals. Our results indicated that both sleep duration and *APOE* genotype independently influence LC-FC in key brain regions involved in cognitive processes, such as the TP and MCC. Importantly, a significant interaction effect was observed between sleep duration and *APOE* genotype on the left LC-FC with PreC, and right LC-FC with MTG, and lOFC. Additionally, mediation analysis revealed that FC between the right LC and the right lOFC, as well as between the left LC and MCC, partially mediated the relationship between age and memory performance. These findings highlight the potential role of both sleep and genetic factors in modulating LC connectivity, which may influence early cognitive changes in AD progression. As such, LC-FC connectivity could serve as a promising therapeutic target for preserving cognitive health in at-risk populations.

### Main effects of sleep duration and *APOE* genotype on the LC-FC network

Our findings revealed that both sleep duration and *APOE* genotype independently influence the LC-FC network in CUOA-Aβ+ individuals. Specifically, short sleep duration was associated with decreased left LC-FC with the TP, and right LC-FC with the TP, MCC, and pons, while the *APOE* ɛ4 genotype also exhibited decreased left LC-FC in the TP and MCC. The TP and MCC are key nodes within emotional and attentional regulation networks and are essential for memory integration and executive function.^37-39^ Previous studies have also manifested altered MCC function in chronic insomnia disorder and changes in LC function in subjective cognitive decline elders.^19,40^ Additionally, our results showed that *APOE* ɛ4 carriers exhibited increased right LC-FC in the SPL and STG, which are involved in sensory integration and spatial processing.^41^ These functions are often compensatory enhanced in *APOE* ɛ4 carriers to counterbalance the risk of AD pathology.^42-44^ Taken our findings align with existing literature, emphasizing the role of both sleep and *APOE* genotype in modulating sensory and emotional function within the LC-FC network. Overall, our findings suggest that even in clinically unimpaired individuals, short sleep duration and the *APOE* ɛ4 carriers are associated with altered LC-FC patterns, potentially predisposing them to cognitive decline.

### Interactive effect of sleep and *APOE* genotype

The interaction between sleep duration and *APOE* genotype on LC-FC was a significant finding in our study. The differential effects observed in regions such as the PreC (left LC-FC network), MTG and lOFC (right LC-FC network) suggest that sleep duration modulates the impact of the *APOE* genotype on LC-FC. Specifically, *APOE* ɛ4 carriers with short sleep duration exhibited increased LC-FC connectivity in the PreC and MTG. These regions are integral to motor planning and memory processing, respectively. The MTG, in particular, has been implicated in semantic memory and language processing, functions that are often compromised in early AD.^45,46^ The increased LC connectivity in these regions may reflect a compensatory mechanism in *APOE* ɛ4 carriers with adequate sleep, potentially supporting resilience in semantic and motor functions during the pre-clinical stage of AD. In contrast, *APOE* ɛ4 carriers with short sleep duration exhibited decreased LC-FC network in the lOFC. The lOFC is essential for decision-making and emotional regulation.^47^ The decreased connectivity in lOFC may reflect a dysfunctional emotional regulation mechanism in *APOE* ɛ4 carriers with inadequate sleep. This finding is consistent with growing evidence that sleep disturbances exacerbate the effects of genetic risk factors on brain function.^11^ These results underscore the importance of considering both genetic and lifestyle factors, such as sleep duration, in understanding AD risk.

### The LC-FC associated cognitive performance in CUOA-aβ+ individuals

Our results also indicate that alterations in LC-FC are linked to cognitive performance, particularly in individuals with short sleep durations. The positive correlation between LC-FC in regions such as the TP and memory performance suggests that the LC network plays a crucial role in maintaining cognitive function, especially in short-sleeping CUOA- Aβ+ individuals. These findings are consistent with previous research showing that the LC, as one of the first regions to exhibit AD pathology, plays a central role in early cognitive decline. These findings highlight the potential of LC-FC as a biomarker for early cognitive decline. Monitoring changes in LC-FC could offer insights into an individual’s risk of cognitive impairment, particular in the context of sleep disturbances in CUOA-Aβ+ individuals. Although the current study found no significant associations between LC-FC connectivity and cognitive assessment in the CUOA-Aβ+ group, this may reflect the influence of cognitive reserve.^19^

### Mediation of LC-FC on the age-related cognitive decline

Our mediation analysis provides further insights into the role of LC-FC in age-related cognitive decline. Age plays a central role in cognitive performance in CUOA-Aβ+ individuals, including general cognition, memory and executive function performance. Notably, the functional connectivity between the right LC and the right lOFC partially positively mediated the association between age and memory performance. The lOFC is involved in decision-making and emotional regulation, functions that are typically impaired with aging.^48^ This aligns with previous research showing that disruptions in the orbitofrontal cortex are associated with age-related decline in decision-making and emotional stability, particularly in memory-intensive tasks.^49^ Similarly, our analysis found FC between the left LC and MCC served as a negative mediator for age-related declines in memory performance. Given the MCC’s role in executive function and cognitive control,^39^ reduced LC-MCC connectivity with advancing age may reflect a loss of functional support for cognitive resilience, predisposing older individuals to memory deficits and executive dysfunction. This mediating effect supports the hypothesis that LC-FC pathways contribute to cognitive aging trajectories and could serve as critical targets for interventions aimed at preserving cognitive health in older adults.

## Limitations

While our study offers important insights into the interaction between sleep, *APOE* genotype, and LC-FC, several limitations must be noticed. First, the cross-sectional design precluding causal inferences regarding interactions among sleep duration, *APOE* genotype, LC-FC alterations, and cognitive trajectories. Second, sleep duration was assessed via self-report, which is subject to recall bias and may not accurately reflect participants’ actual sleep patterns. Future studies should employ objective sleep metrics such as sleep quality or sleep efficiency, measured by polysomnography or actigraphy, to validate these findings. Third, as an exploratory analysis, these correlative analyses and mediation models did not employ multiple comparison correction, which increases Type I error risk. Future confirmatory studies should apply appropriate corrections. Fourth, although we controlled for several demographic variables, including age, sex, and education, other potential confounders, such as physical activity, diet, and comorbidities, were not considered in this study. Finally, our study focused on a specific population of the CUOA-Aβ+ individuals. While this population is at high risk for AD, our findings may not be generalizable to other populations, such as those with different levels of amyloid burden levels or individuals who have already developed mild cognitive impairment or dementia. Future studies should examine the generalizability of these findings in more diverse populations.

## Conclusion

This study provides novel insights into how sleep duration and *APOE* genotype independently and interactively affect LC-FC in CUOA-Aβ+ individuals. We found that both short sleep duration and *APOE* ɛ4 genotype were associated with altered LC-FC in regions important for emotional regulation, sensory integration, and executive function. Mediation analysis further suggests that the interaction between sleep duration and *APOE* genotype influences LC-FC connectivity, mediating the relationship between age and cognitive decline. These results underscore the potential of LC-FC as a biomarker for early cognitive decline and emphasize the need to consider genetic and lifestyle factors in Alzheimer’s prevention and management.

## Supplementary Material

fcaf341_Supplementary_Data

## Data Availability

Anonymized data are publicly available to any qualified investigator through the LONI website (ida.loni.usc.edu/login.jsp?project=A4).
