# Supplementary material for: APOE genotype modulates the impact of sleep duration on locus coeruleus functional connectivity in pre-clinical Alzheimer’s disease
Source: Brain Commun. 2025 Sep 9;7(5):fcaf341. doi: 10.1093/braincomms/fcaf341 (PMC12455407; doi:10.1093/braincomms/fcaf341)
Supplement: fcaf341_Supplementary_Data [file fcaf341_supplementary_data.docx]

***APOE* genotype modulates the impact of sleep duration on locus coeruleus functional connectivity in preclinical Alzheimer’s disease**

**Supplementary Table 1 Post-hoc analysis of ANCOVA results for demographic/cognitive data between *APOE* ε4/ε4 and ε3/ε4 groups**

|  | *APOE* ε3ε4 (n=348) | *APOE* ε4ε4 (n=54) | *T/X^2^(p)* |
| --- | --- | --- | --- |
| Sleep Duration(Normal/Short) | 249/99 | 42/12 | 0.91(0.34)* |
| Sex(F/M) | 206/142 | 35/19 | 0.62(0.43)* |
| Age(yrs) | 71.53±4.47 | 69.56±3.57 | 3.10(0.002) |
| Education (yrs) | 16.46±2.76 | 16.19±2.48 | 0.69(0.49) |
| MMSE | 28.59±1.35 | 28.78±1.31 | -0.98(0.33) |
| PACC | -0.68±2.73 | -0.13±2.83 | -1.36(0.18) |
| DSST | 42.1±9.18 | 44.26±9.09 | -1.61(0.11) |
| FCSRT | 75.23±6.25 | 75.04±6.53 | 0.21(0.84) |
| LMDR | 11.27±3.27 | 11.87±3.68 | -1.24(0.22) |
| ICV (ml) | 1536.79±147.429 | 1509.75±150.625 | 1.24(0.21) |

Notes: *， Chi-square test; Abbreviations: APOE, Apolipoprotein E; PACC, Preclinical Alzheimer’s Cognitive Composite; DSST, Digit Symbol Substitution Test; FCSRT, Free and Cued Selective Reminding Test; LMDR, Logical memory delayed Recall; MMSE, Mini-Mental State Examination; ICV, Intracranial Volume.

**Supplementary Table 2 Post-hoc analysis of ANCOVA results for neuroimaging data between *APOE* ε4/ε4 and ε3/ε4 groups**

|  | | *APOE* ε3ε4 (n=348) | *APOE* ε4ε4 (n=54) | *T(p)* |
| --- | --- | --- | --- | --- |
| Left LC-FC network | | | | |
| Main effect of Sleep | Right TP | 0.083±0.004 | 0.073±0.01 | 0.289(0.773) |
| Main effect of *APOE* | Left MCC | 0.101±0.005 | 0.093±0.013 | 0.467(0.642) |
|  | Right TP | 0.078±0.004 | 0.079±0.011 | -0.208(0.835) |
| Interactive effect of Sleep × *APOE* | Right PreC | 0.106±0.006 | 0.09±0.012 | 0.527(0.598) |
| Right LC-FC network | | | | |
| Main effect of Sleep | Right TP | 0.085±0.005 | 0.075±0.01 | -0.594(0.553) |
|  | Right Pons | 0.125±0.007 | 0.107±0.015 | 0.736(0.464) |
|  | Bilateral MCC | 0.098±0.005 | 0.075±0.01 | -0.787(0.432) |
| Main effect of *APOE* | Left STG | 0.09±0.005 | 0.101±0.014 | 0.101±0.014 |
|  | Right SPL | 0.087±0.005 | 0.076±0.01 | 0.383(0.702) |
| Interactive effect of Sleep × *APOE* | Left MTG | 0.088±0.005 | 0.071±0.01 | 0.871(0.384) |
|  | Right lOFC | 0.082±0.004 | 0.084±0.011 | 0.012(0.99) |

Abbreviations: LC-FC, locus coeruleus functional connectivity; TP, temporal pole; MCC, middle cingulate cortex; PreC, precentral gyrus; STG, superior temporal gyrus; SPL, superior parietal lobule; MTG, middle temporal gyrus; lOFC, lateral orbitofrontal cortex.


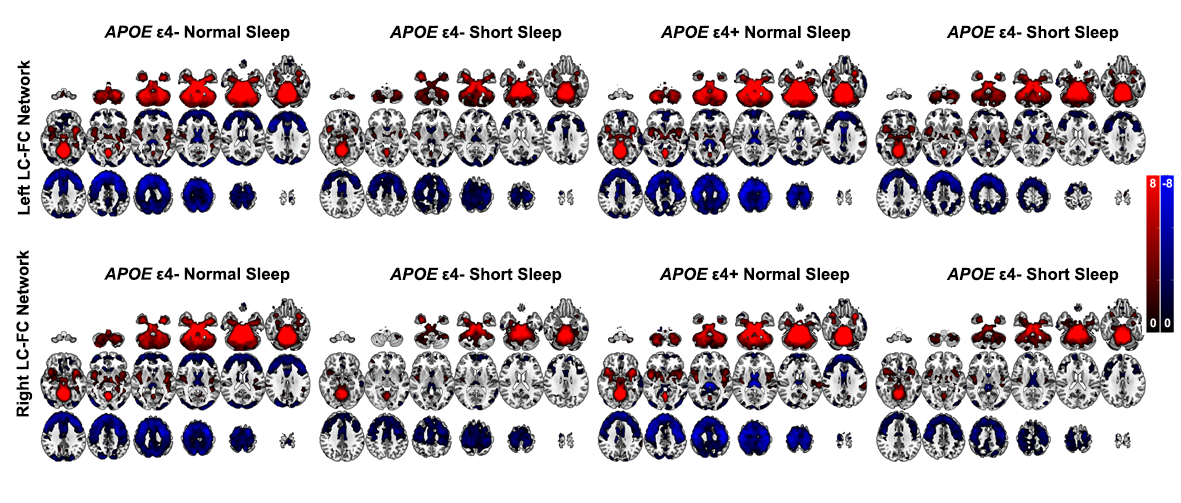


**Supplementary Figure 1 - The left and right LC-FC network patterns for each group (one sample t-test, voxel *p* < 0.05, uncorrected).** *APOE* ε4- Normal Sleep group, n = 210**;** *APOE* ε4- Short Sleep group, n = 80; *APOE* ε4+ Normal Sleep group, n = 291; *APOE* ε4- Short Sleep group, n = 111. The color bar means the T value. Abbreviations: LC-FC, locus coeruleus functional connectivity.


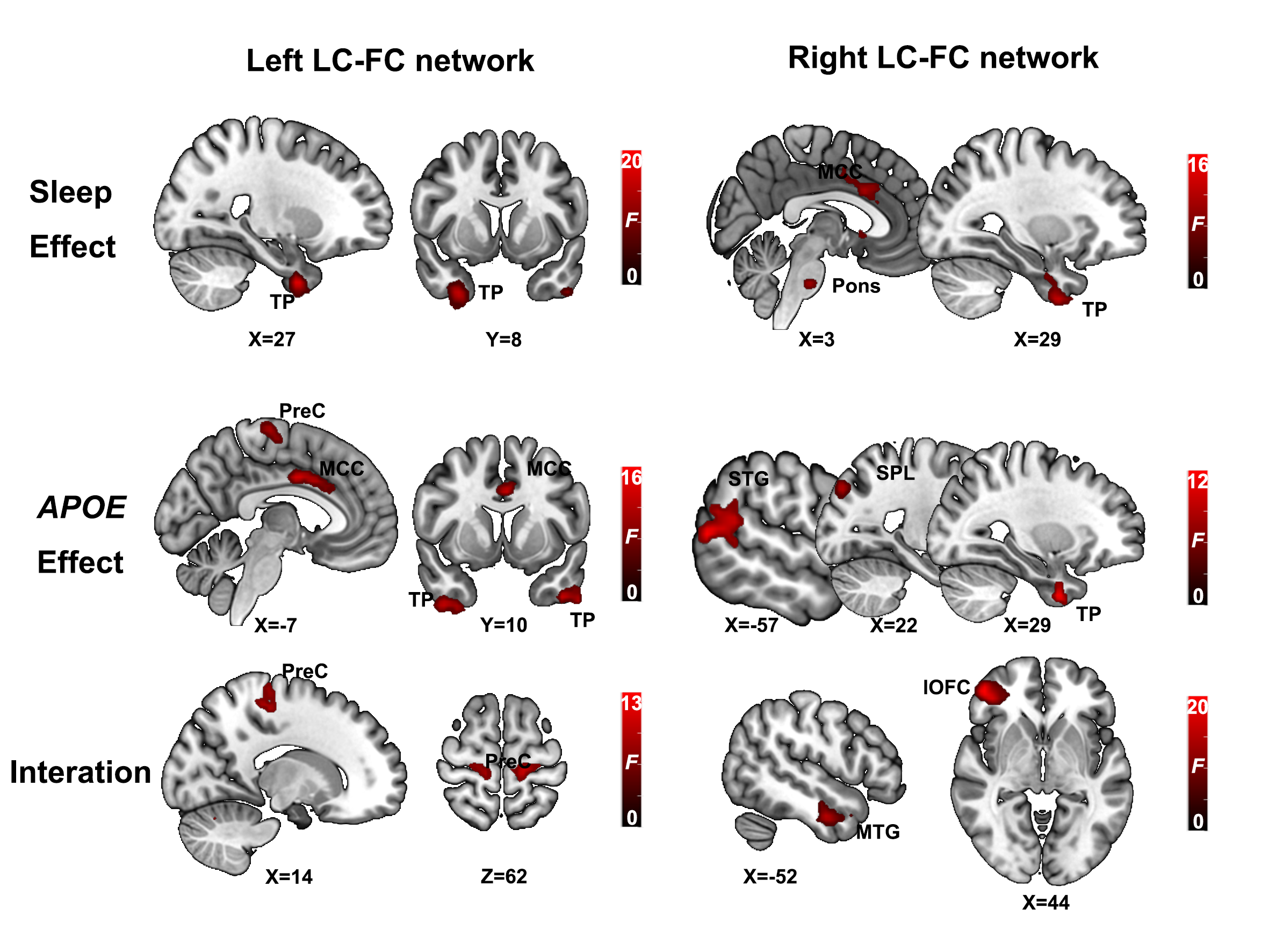


**Supplementary Figure 2 - The Sensitivity neuroimaging ANCOVA analysis results of using 3mm Gaussian Kernel smoothing in the all participants (*p* < 0.005, *α* < 0.01, GRF correction, n = 692).** The color bar means the *F* value. Abbreviations: LC-FC, locus coeruleus functional connectivity; TP, temporal pole; MCC, middle cingulate cortex; PreC, precentral gyrus; STG, superior temporal gyrus; SPL, superior parietal lobule; MTG, middle temporal gyrus; lOFC, lateral orbitofrontal cortex.
